# Optimizing precision rhinoplasty: comprehensive preoperative planning with nasal computed tomography for functional and aesthetic enhancement

**DOI:** 10.1186/s40902-024-00423-y

**Published:** 2024-03-25

**Authors:** Pawel Szychta

**Affiliations:** 1 Dr Szychta Clinic chirurgiaplastyczna.pl, Gdansk, Poland; 2 Department of Surgical Oncology and Breast Diseases, Polish Mother’s Memorial Hospital — Research Institute, Lodz, Poland

**Keywords:** Rhinoplasty, Preoperative planning, Nasal computed tomography, Functional enhancement, Aesthetic optimization, Surgical modifications, Nasal anatomy

## Abstract

**Background:**

The evolving field of rhinoplasty increasingly recognizes the importance of clinical expertise over routine preoperative nasal computed tomography (CT) for planning surgical interventions. This study evaluates the clinical utility of preoperative nasal CT in enhancing the precision of open structured rhinoplasty, focusing on both functional and aesthetic outcomes without compromising patient safety through unnecessary radiation exposure. The study aimed to assess the impact of preoperative nasal CT on surgical planning and intraoperative maneuvers.

**Methods:**

A prospective cohort study involved patients undergoing open structured rhinoplasty with or without preoperative nasal CT. Participants were divided into a study group, receiving preoperative nasal CT, and a control group, undergoing rhinoplasty without such imaging. Surgical modifications were tailored based on CT findings, with outcomes evaluated through postoperative nasal airflow and aesthetic satisfaction.

**Results:**

The study included 205 patients in the CT group and 514 in the control group, with comparable demographics. The CT group demonstrated significant improvements in nasal breathing and higher aesthetic satisfaction postoperatively, with a notable decrease in the NOSE score and an increase in the ROE score compared to the control group. Minor complications were observed in a small percentage of the CT group, showcasing a nuanced approach to addressing individual anatomical variations.

**Conclusions:**

Preoperative nasal CT in open structured rhinoplasty significantly enhances surgical precision, optimizing functional and aesthetic outcomes. This study underscores the utility of preoperative CT in individualized surgical planning, suggesting its pivotal role in the advancement of rhinoplasty practices. Future research should explore long-term benefits and further validate these findings across diverse populations.

## Introduction

In the contemporary landscape of rhinoplasty, an increasing body of evidence supports the feasibility of conducting this intricate surgical procedure with precision and safety, relying on thorough preoperative clinical analysis while foregoing the routine use of preoperative nasal computed tomography (CT). This paradigm shift underscores a strategic approach where the clinical acumen, derived from a detailed physical examination and patient history, suffices in mapping out the surgical plan. This methodology not only streamlines the preoperative phase by eliminating the need for extensive radiographic imaging but also highlights the clinical utility and rationale behind minimizing patient exposure to unnecessary radiation. By leveraging clinical expertise and judicious use of diagnostic tools, surgeons can effectively address both functional and aesthetic nasal concerns, thereby optimizing patient outcomes with a focus on safety, efficacy, and patient-centric care.

Here, rhinoplasty, a surgical intervention addressing both functional and aesthetic aspects of the nose, continues to evolve with an increasing emphasis on precision and individualized patient outcomes. In the pursuit of optimizing precision rhinoplasty, preoperative planning plays a pivotal role in guiding surgical maneuvers [1]. This study explores the clinical applicability and the direct impact of incorporating preoperative nasal computed tomography (CT) into the planning process for open structured rhinoplasty [2]. Integration of preoperative nasal CT as an integral component of planning allows for a comprehensive understanding of the anatomical intricacies of the nose and nasal cavity [3]. Advantages of preoperative nasal CT include enhanced visualization options of the three-dimensional imaging provided by nasal CT, which facilitates an in-depth assessment of the nasal anatomy, allowing for a detailed exploration of the nasal septum, turbinates, nasal dorsum, nasal tip, nasal bones, nasal alae, soft tissues/skin, paranasal sinuses, nasopharynx, and olfactory structures [4]. Preoperative nasal CT enables accurate mapping of nasal structures, providing a road map for individualized surgical planning [5]. By focusing on the nasal septum, turbinates, and sinus structures, preoperative nasal CT aids in addressing functional issues, contributing to improved postoperative nasal airflow [6]. Visualizing the nasal dorsum, tip, bones, and alae in detail allows for a more refined and individualized approach to achieving optimal aesthetic outcomes [7]. Therefore, the detailed insights gained from preoperative nasal CT can potentially guide the customization of surgical maneuvers, allowing for a nuanced approach to address specific anatomical variations and concerns [8].

This study aims to delineate the clinical utility of preoperative nasal CT in optimizing precision rhinoplasty, emphasizing its role in refining surgical planning and modifying intraoperative maneuvers, for enhanced surgical outcomes. Through a comparative analysis with the control group, we seek to underscore the tangible benefits of incorporating this advanced imaging modality in enhancing both functional and aesthetic outcomes in open structured rhinoplasty.

## Methods

The study was conducted in accordance with ethical standards and was approved by the Ethical Committee of the Polish Mother’s Memorial Hospital — Research Institute in Lodz in Poland. The study adhered to ethical principles outlined in the Declaration of Helsinki. Patient confidentiality was maintained, and data were anonymized during analysis to ensure privacy.

The study enrolled patients who underwent open structured rhinoplasty for a spectrum of functional and aesthetic nasal issues. The cohort included individuals with a range of nasal issues, such as septal deviations, turbinate hypertrophy, nasal tip deformities, and nasal bone irregularities. The exclusion criteria were any previous nasal surgeries or other procedures in the nasal area, chronic diseases which could affect the outcome and post-cleft deformities, to minimize bias. Following early inclusion criteria to the study, patients were included in the study group only when they consented for the preoperative CT scanning; otherwise, they were considered as a control group following randomization. The study group underwent preoperative nasal CT as an integral component of the preoperative planning process. High-resolution CT scans were obtained in axial and coronal planes to comprehensively visualize the nasal anatomy. CT imaging was performed using a standardized protocol to visualize specific anatomical areas critical for rhinoplasty, including the nasal septum, turbinates, nasal dorsum, nasal tip, nasal bones, nasal alae, soft tissues/skin, paranasal sinuses, nasopharynx, and olfactory structures. The control group consisted of patients who underwent rhinoplasty without preoperative nasal CT. Randomization for the control group was employed to either assign patients with no preoperative CT to the control group or to exclude from the study. Randomization was utilized to ensure a representative sample that paralleled the demographics of the study group.

**Fig. 1:**
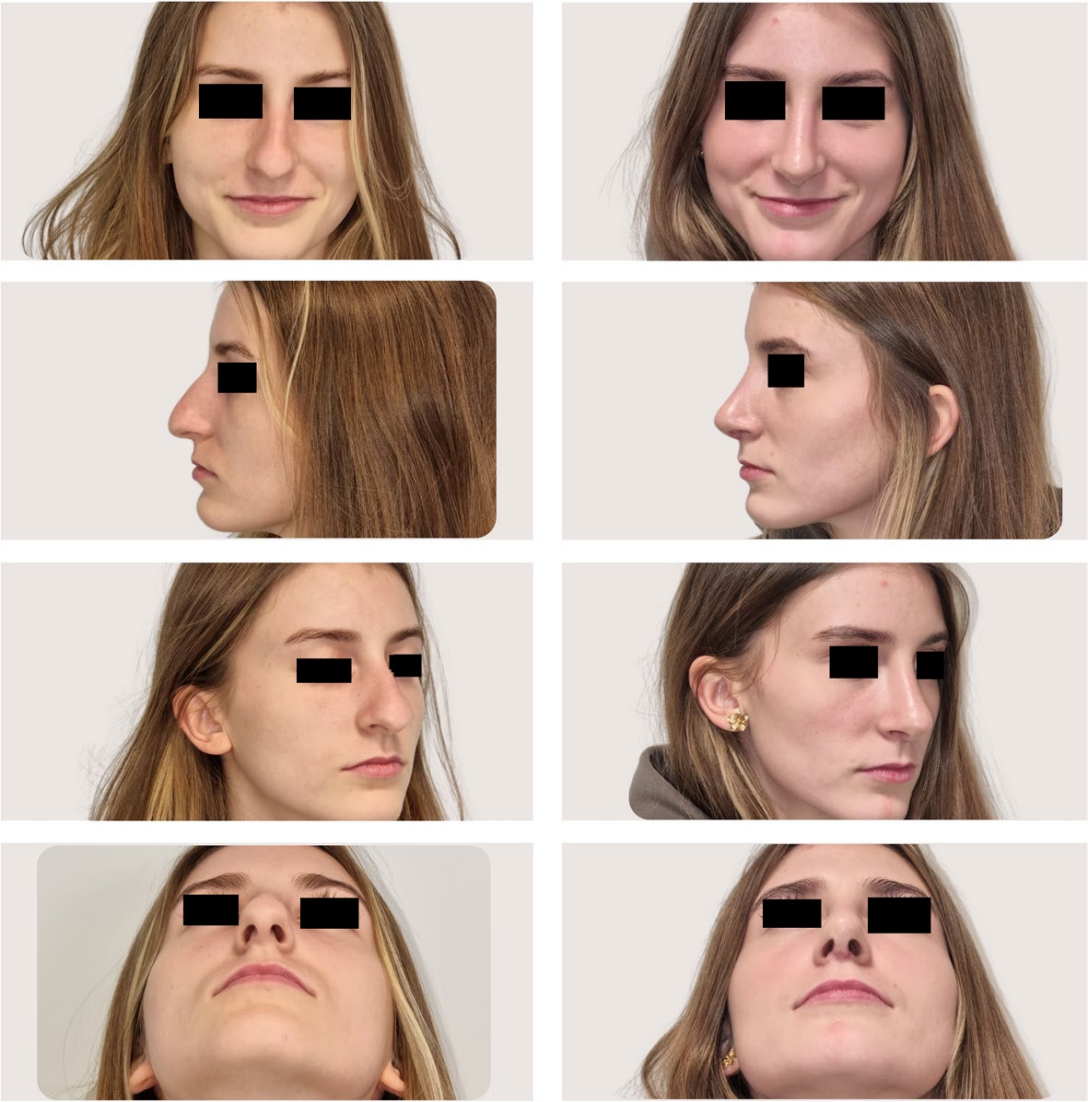
CT-assisted precision rhinoplasty in patient A desiring natural feminine harmonious aesthetic outcome

**Fig. 2:**
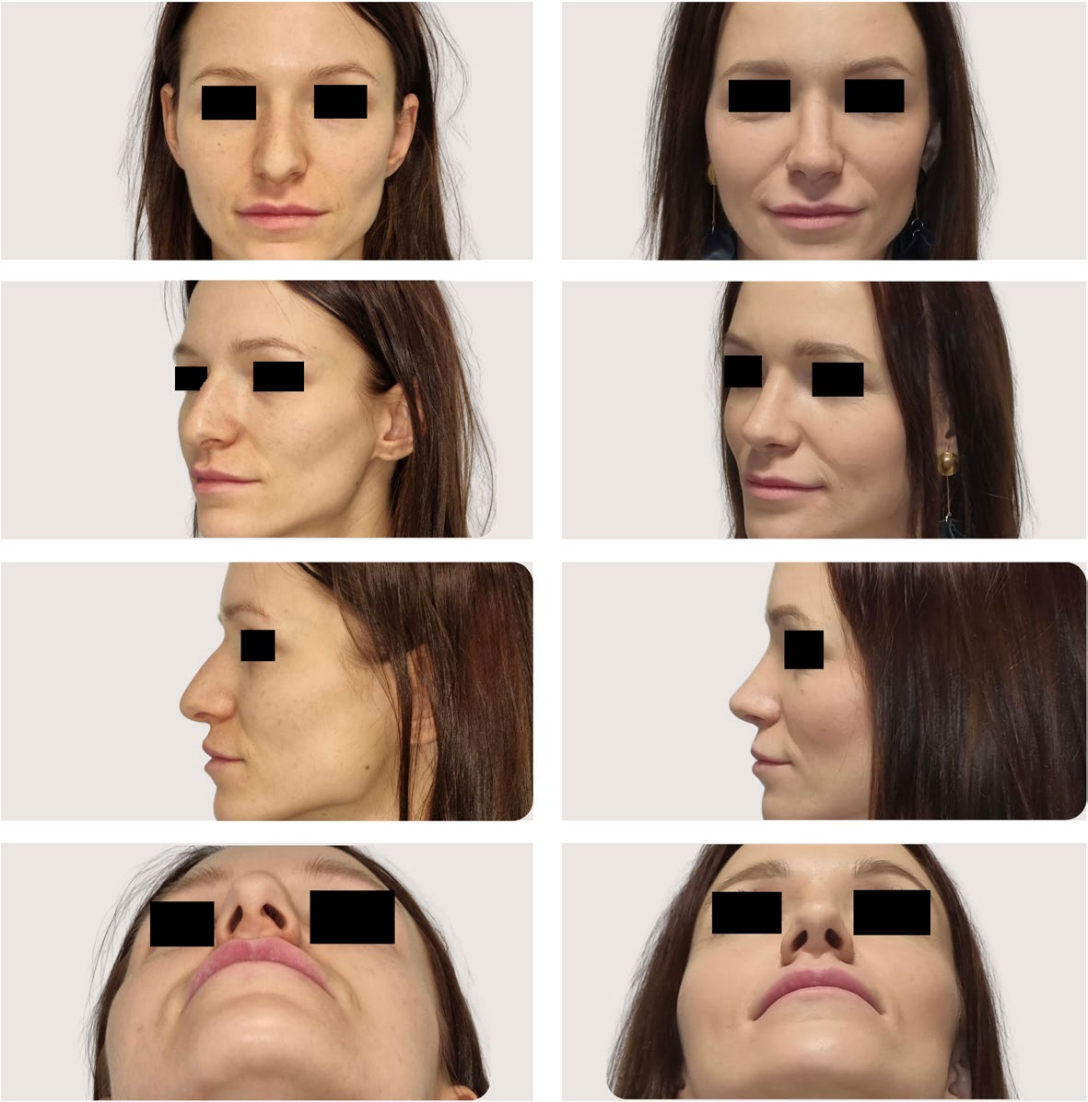
CT-assisted precision rhinoplasty in patient B desiring sensual feminine aesthetic outcome

Preoperative nasal CT included detailed anatomical assessment, where three-dimensional imaging provided comprehensive insights into the nasal anatomy, aiding in the identification of specific anatomical variations and concerns. An experienced rhinoplasty surgeon analyzed the CT images. Anatomical variations and pathologies in the nasal region were meticulously assessed. The CT findings were incorporated into the preoperative planning, informing decisions on specific surgical maneuvers (Figs. 1, 2).

Experienced rhinoplasty surgeon performed all procedures using an open structured approach [9–11]. Postoperative detailed examination on follow-up consultation was done to validate the accuracy of preoperative planning and assess the impact of wide range of detailed intraoperative modifications on the nasal anatomy, for individualized clinical implications.

Primary outcome measures included postoperative functional improvement, assessed through objective measures of nasal airflow, and aesthetic outcomes evaluated by both surgeons and patients [12]. Nasal symptoms were evaluated using a validated, disease-specific, quality-of-life instrument. The Nasal Obstruction Symptom Evaluation (NOSE) questionnaire was administered to participants at baseline and 3 months postoperatively [12]. Clinical effect of surgical intervention was assessed at 3 months postoperatively as a change in a NOSE multiplied scale score ranging from 0 to 100, with the higher score suggesting the more severe obstruction. Evaluation of patient aesthetic satisfaction was undertaken including Rhinoplasty Outcome Evaluation (ROE) questionnaire, with multiplied score ranging from 0 to 100 [13].

Descriptive statistical methodologies were utilized to encapsulate the demographic and clinical attributes of the patient cohort. Paired *t*-tests were applied to evaluate the alterations in the NOSE and ROE scores preoperatively and at a 3-month postoperative interval, thereby quantifying the significance of functional and aesthetic enhancements observed.

## Results

The prospective cohort included 205 patients (76.59% women, 23.41% men) who underwent open structured rhinoplasty for a spectrum of functional and aesthetic nasal concerns, based on the preoperative nasal CT. The control group comprised a randomized selection of 514 patients (83.85% women, 16.15% men) who underwent rhinoplasty without preoperative nasal CT. Demographic characteristics, including age, gender distribution, and primary nasal concerns, were comparable between the study and control groups, ensuring a representative sample (Table 2).

The following anatomical details of the external nose and nasal cavity were accessible through computed tomography (CT) imaging, influencing preoperative planning and subsequent surgical maneuvers: nasal septum (detection of septal deviations, spurs, and deviations in the septal cartilage; identification of septal thickness variations), turbinates (visualization of turbinate hypertrophy and anatomical variations; assessment of the size, shape, and configuration of the inferior and middle turbinates), nasal dorsum (detailed imaging of the nasal dorsum, including bony and cartilaginous components; identification of dorsal humps, depressions, and deviations), nasal tip (visualization of the nasal tip structure, including the alar cartilages, assessment of tip projection, rotation, and symmetry), nasal bones (detailed imaging of the nasal bones, aiding in the assessment of fractures or deviations), nasal alae (detection of asymmetry or irregularities in the nasal alae; visualization of the nasal alar contour), soft tissues and skin (assessment of skin thickness and soft tissue characteristics, which can impact surgical planning, especially in rhinoplasty), paranasal sinuses (evaluation of paranasal sinuses — such as the maxillary, ethmoid, frontal, and sphenoid sinuses — for potential anatomical variations or pathology), nasopharynx (visualization of the nasopharynx, which may influence surgical decisions related to airflow and function), and olfactory structures (though not always the primary focus, CT may provide information on the olfactory structures within the nasal cavity) (Table 1) [14–16].

**Table 1:** Anatomical details detected by nasal computed tomography in optimizing precision rhinoplasty, clinical applicability and surgical modificationsAnatomical feature

Anatomical Feature	Preoperative finding	Measurements required	Surgical modification	Specific measurements required	Clinical Outcome
Nasal septum	Deviations, spur	Septum thickness, deviation angle	Straightening, reinforcement	Septum thickness, deviation angle	Improved airflow, symmetry
Nasal bones	Deviations, fractures	Bone length, deviation degree	Osteotomy, realignment	Bone length, deviation degree	Stabilized structure, aesthetics
Nasal cartilages	Weak tip support	Cartilage strength, tip projection	Tip refinement, support	Cartilage strength, tip projection	Enhanced tip definition
Turbinates	Hypertrophy	Turbinate size, degree of hypertrophy	Reduction, resection	Turbinate size, degree of hypertrophy	Reduced obstruction, better airflow
Nasal soft tissues	Irregularities	Skin thickness, soft tissue volume	Camouflage, smoothing	Skin thickness, soft tissue volume	Uniform contour, aesthetics
Alar base and nasal ligaments	Wide alar base	Alar base width, ligament positions	Base reduction	Alar base width, ligament positions	Narrowed base, improved proportions
Nasal dorsum and radix	Dorsal hump	Dorsum height, radix depth	Dorsal reduction, augmentation	Dorsum height, radix depth	Smooth dorsal aesthetic line
Nasal tip and columella	Poor definition	Tip projection, columella length	Tip definition, support	Tip projection, columella length	Defined tip, better projection
Nasal airways	Narrow valves	Valve angle, airway diameter	Valve widening	Valve angle, airway diameter	Improved nasal breathing

**Fig. 3:**
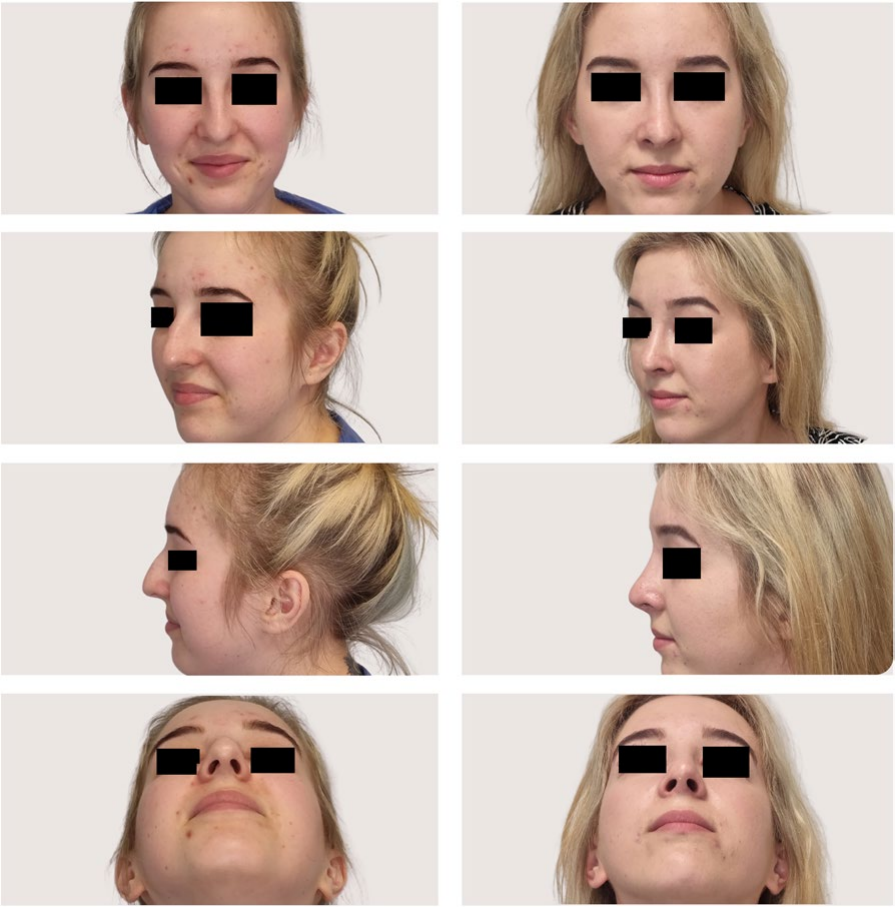
CT-assisted precision rhinoplasty in patient C desiring classical subdued aesthetic outcome

**Fig. 4:**
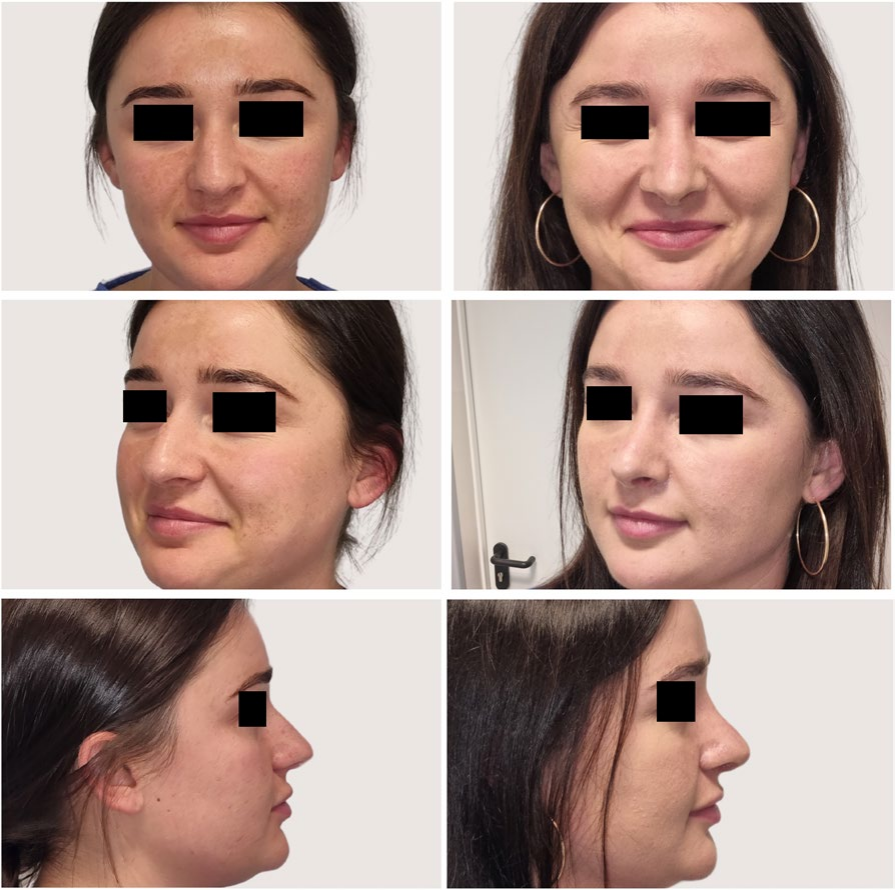
CT-assisted precision rhinoplasty in patient D desiring natural feminine harmonious aesthetic outcome in the nose with “thick” skin

As analyzed retrospectively in the analysis phase of the study, intraoperative several modifications to surgical maneuvers dedicated to the specific anatomical variations were made based on the individualized wide range of preoperative CT findings (Figs. 3, 4). Generally, surgical modifications in the study group, tailored according to preoperative CT findings, included adjustments in septoplasty, turbinate management, dorsal contouring, tip refinement, bone modifications, alar symmetry considerations, soft tissue adjustments, sinus-related modifications, and improvements in nasal airflow. Specific areas of focus included tailored septoplasty techniques, refinement of turbinate management, precise alterations to the nasal dorsum, and nuanced adjustments to the nasal tip, involving alterations in the following procedures: septoplasty (precise adjustments based on the detected septal deviations, thickness variations, and anomalies), turbinoplasty (tailored turbinate management based on CT findings, addressing hypertrophy and variations), dorsal contouring (informed modifications to address dorsal humps, depressions, or deviations detected on CT), nasal tip surgery (nuanced adjustments to achieve the desired tip projection, rotation, and symmetry based on CT-guided insights), and osteotomies (if nasal bone deviations or fractures are present, osteotomies may be modified accordingly).

**Fig. 5:**
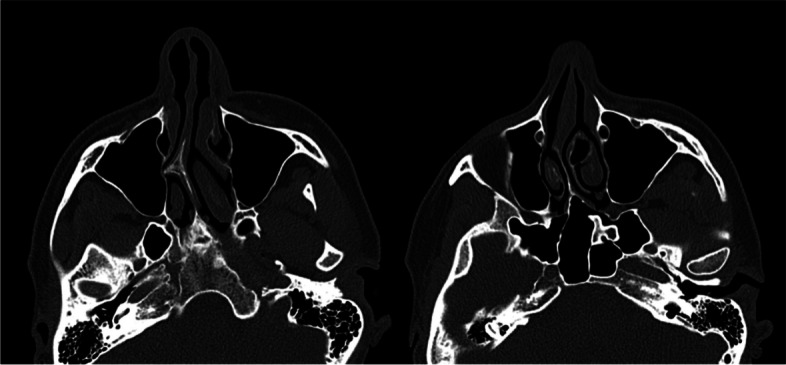
CT of the external nose and nasal cavity prior to CT-assisted precision rhinoplasty of patient B who initially appeared for rhinoplasty solely for aesthetic indications, depicting (**a**) large nasal spur, hypertrophic left lower and middle turbinates, (**b**) narrow internal nasal valves, high septal deviation, left concha bullosa, thick soft tissues

**Fig. 6:**
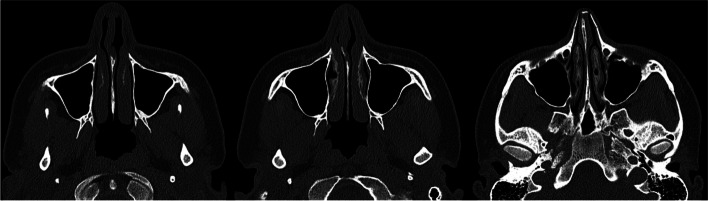
CT of the external nose and nasal cavity prior to CT-assisted precision rhinoplasty of Patient D who initially appeared for rhinoplasty solely for aesthetic reasons, depicting the following: (**a**) septal deviation, hypertrophic lower turbinates with bilateral airway obstruction, (**b**) very narrow right and narrow left internal nasal valve with chondral and bony septal deviation, **c** high deviation of the bony septum with double-layer widened septal ethmoid bone, left concha bullosa

**Fig. 7:**
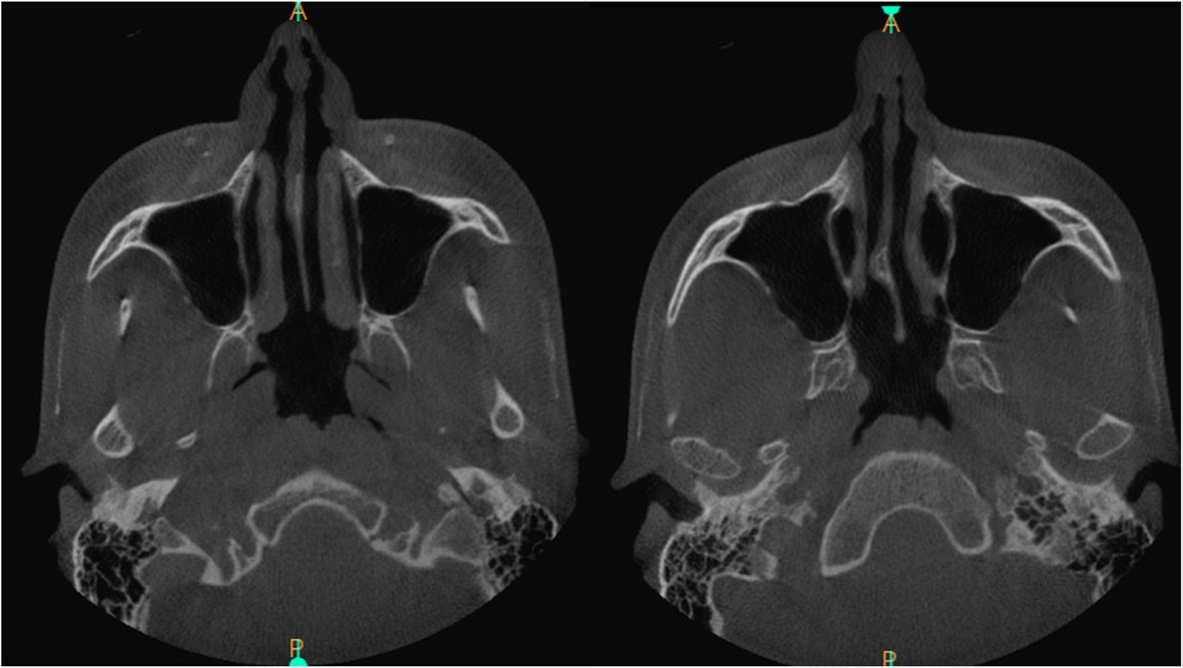
Computed tomography of the nasal cavity prior to CT-assisted precision rhinoplasty, depicting the distortions of the nasal tip, weak external nasal valves, collapsed internal nasal valves, septal spur

**Fig. 8:**
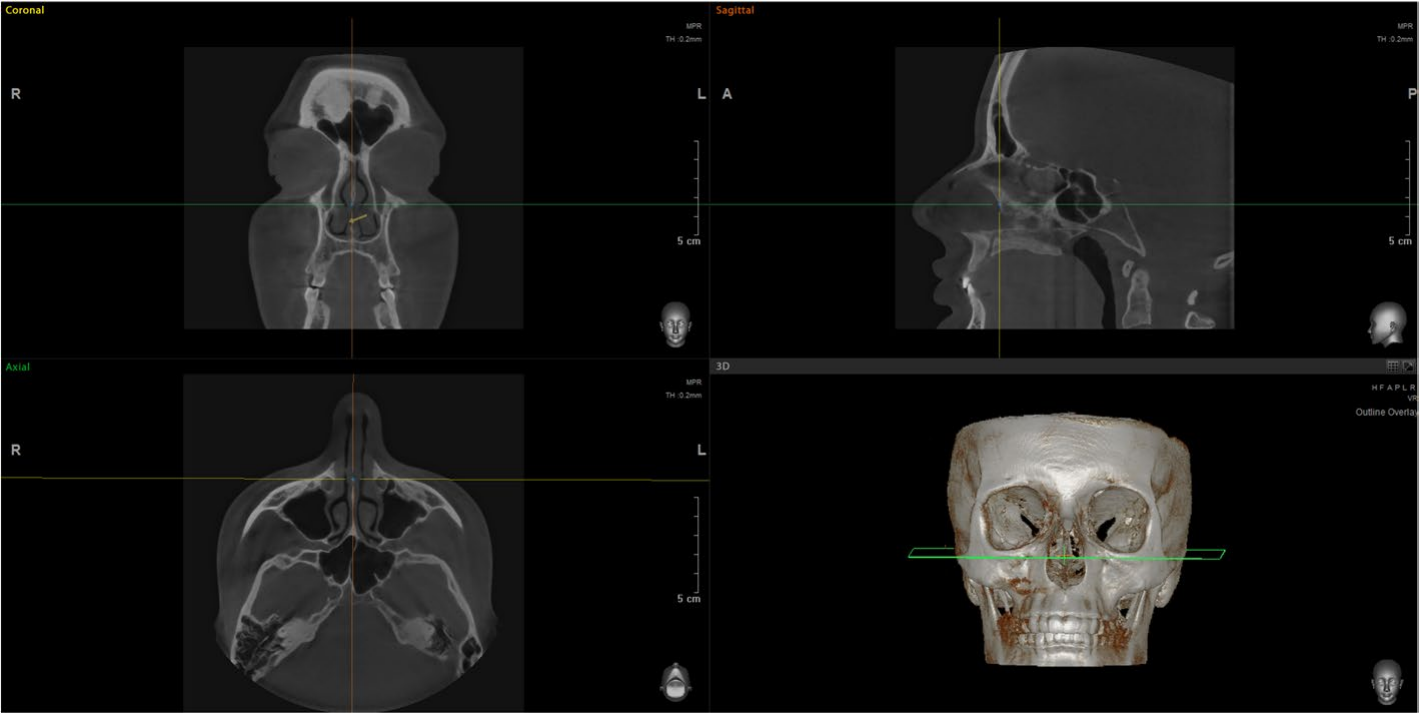
Computed tomography of the nasal cavity prior to CT-assisted precision rhinoplasty, depicting the distortions of the narrow internal nasal valves, hypertrophic turbinates, soft tissues thickening of sidewalls and septum, dimensions and relationships between chondral septum, ethmoid bone, and vomer; 3D reconstruction is extremely useful for precise understanding of anatomy for individualized surgical plan and predictable maneuvers focused on specific nuances

The study group exhibited refined surgical outcomes attributed to the incorporation of preoperative nasal CT. Enhanced precision in addressing specific anatomical variations resulted in improved functional and aesthetic outcomes (Figs. 5, 6, 7, 8). The control group, undergoing rhinoplasty without preoperative nasal CT, demonstrated satisfactory outcomes; however, the degree of precision in addressing individualized anatomical variations was comparatively limited (Table 2). In the control group, a moderate improvement was seen in NOSE questionnaire, with a decrease of 46.65 points from the baseline of 84.82 (*p* < 0.05). In the aesthetic context of analysis, ROE questionnaire revealed relatively high patient satisfaction with the preoperative score of 24.44 and the postoperative score of 72.71 (*p* < 0.05). In 89% patients, their appearance was still rated as improved; however, 3% were relatively satisfied about the appearance, and 8% felt they did not benefit from any improvement. In the study group, a significant general improvement was found in nasal breathing, with a mean decrease of 78.55 points from the preoperative NOSE score at the 3-month assessment when compared with the preoperative baseline of 91.45 (*p* < 0.05). When taking aesthetic assessment into consideration, ROE questionnaire revealed very high patient satisfaction with score changing from preoperative 21.76 to postoperative level of 88.20 (*p* < 0.05). In 96% patients, their appearance was rated as improved, 2% were relatively satisfied about the appearance, and 2% felt they did not benefit from any improvement. Statistical analyses were conducted to assess the significance of the observed differences between the study and control groups. The study group consistently demonstrated statistically significant improvements in outcomes related to both functional (*p* < 0.05) and aesthetic parameters (*p* < 0.05).

**Table 2:** Comparative analysis of rhinoplasty outcomes in study and control groups

Group of patients	Parameter	Before/after rhinoplasty	Median	SD	Mean	Min	Max
Study group	Age		36	8.45	33.40	18	45
	NOSE	Before	92	6.65	91.45	72	100
		After	12	13.96	12.90	0	64
	ROE	Before	23	13.07	21.76	2	46
		After	100	15.87	88.20	39	100
Control group	Age		32	9.15	32.61	18	49
	NOSE	Before	88	15.20	84.82	32	100
		After	36	8.68	38.17	16	56
	ROE	Before	25	13.66	24.44	4	50
		After	73	9.81	72.71	58	91

*NOSE* Nasal Obstruction Symptom Evaluation questionnaire, *ROE* Rhinoplasty Outcome Evaluation questionnaire, *SD* standard deviation

Complications were monitored postoperatively, and no major complications were reported in the study group. Minor complications, such as tip asymmetry (*n* = 3), too defined tip (*n* = 2), dorsal irregularity (*n* = 1), and residual septal deviation (*n* = 1), were observed in a small percentage of cases. The resulting overall complication rate in the study group was at level of 3.41%, consistent with rates reported in the literature. Conversely, still low rate of complications was encountered, but they were statistically more frequent than the study group (*p* < 0.05), including the following: inadequate tip correction (*n* = 11), dorsal irregularities (*n* = 10), breathing difficulties requiring reoperation (*n* = 3), and residual septal deviation requiring reoperation (*n* = 1), constituting the overall complication rate at level of 4.96%.

## Discussion

Rhinoplasty, a complex surgical intervention addressing both functional and aesthetic concerns, is continually evolving to optimize precision and individualized outcomes [10]. This prospective cohort study aimed to evaluate the clinical applicability of preoperative nasal computed tomography in optimizing precision rhinoplasty for both functional and aesthetic enhancements, focusing on refining surgical planning and modifying specific intraoperative maneuvers. The study sought to identify specific areas of the nose and nasal cavity where intraoperative maneuvers were modified based on preoperative nasal CT findings. Visualization of the nasal dorsum, tip, bones, and alae allowed for a nuanced and individualized approach to aesthetic refinement [17–19]. Therefore, precise mapping of nasal structures resulted directly in the CT-guided, tailored surgical planning, strictly based on the unique characteristics of each patient. Evaluation of the nasal septum, turbinates, and sinuses facilitated a focused approach to address functional concerns, for the enhanced postoperative nasal airflow [20].

Preoperative nasal computed tomography (CT) played a pivotal role in identifying specific anatomical variations and determining the precise measurements required for each intervention. For instance, in cases involving the nasal septum, deviations and spurs were quantitatively assessed with measurements of septum thickness and deviation angles guiding the tailored application of septal cartilage grafts. Similarly, for nasal bone modifications, the length and degree of deviations were measured, informing the use of radix grafts for precise realignment. Cartilage strength and tip projection measurements were critical in cases requiring nasal cartilage adjustments, where alar rim grafts and spreader grafts were utilized based on specific cartilage strength and projection metrics. The size and degree of turbinate hypertrophy were quantified, directing the application of valve support grafts. Skin thickness and soft tissue volume measurements facilitated the strategic use of camouflage grafts in addressing nasal soft tissue irregularities. Alar base width and ligament positions were also measured to guide alar base reduction grafts for improved nasal proportions. Measurements of dorsum height and radix depth were essential in dorsal onlay graft applications for dorsal contouring. Tip projection and columella length measurements informed the use of tip grafts and columellar struts for nasal tip refinement. Lastly, valve angle and airway diameter measurements were crucial in planning spreader grafts for nasal airway improvement. These specific measurements, derived from preoperative CT scans, enabled a highly individualized surgical plan for each patient, ensuring precision in addressing both functional and aesthetic nasal concerns.

Several clinical implications can be drawn from the results of the study, as the observed advantages of preoperative nasal CT were diverse, multifactorial, and individualized. In general, the implementation of preoperative nasal CT allowed for advanced three-dimensional visualization of the external nose and nasal cavity. Similarly to the previous reports, the imaging modality facilitated detailed mapping of critical anatomical structures, encompassing the nasal septum, turbinates, nasal dorsum, nasal tip, nasal bones, nasal alae, soft tissues/skin, paranasal sinuses, nasopharynx, and olfactory structures [2–6]. This comprehensive mapping provided a detailed roadmap for surgical planning, aiding in the identification of specific anatomical variations.

One of the key advantages observed was the ability to tailor surgical planning based on individual patient anatomy. The study group exhibited modifications in intraoperative maneuvers guided by preoperative nasal CT findings. Specific areas of modification included septoplasty, turbinoplasty, dorsal contouring, tip refinement, nasal bone adjustments, alar symmetry considerations, soft tissue adjustments, and sinus-related modifications. This tailored approach allowed for a more precise and individualized response to each patient’s unique nasal anatomy, addressing both functional and aesthetic concerns with a higher degree of accuracy.

Functional concerns, such as nasal septum deviations and turbinate hypertrophy, were addressed with a focused approach, contributing to enhanced postoperative nasal airflow. Aesthetic refinements, including dorsal contouring, tip refinement, and alar symmetry considerations, were guided by preoperative nasal CT, resulting in more nuanced and satisfactory outcomes.

The study group, benefiting from preoperative nasal CT, consistently demonstrated refined surgical outcomes. The precise identification of anatomical variations and subsequent modifications in surgical maneuvers contributed to improved functional and aesthetic outcomes. While the control group exhibited satisfactory outcomes, the degree of precision in addressing individualized anatomical variations was comparatively limited. However, the generalized clinical implications have been summarized in Table 1. This highlights the added value of preoperative nasal CT in refining surgical approaches and optimizing outcomes.

On top of the high range of anatomical variations of the nasal skeleton to be considered prior to rhinoplasty, the outcomes of the procedure are also influenced by a multifaceted interplay of other factors including gender, age, anatomical variances, and systemic diseases. These elements introduce a significant degree of variability in patient responses to surgical interventions, thereby augmenting the complexity of adopting a one-size-fits-all approach in surgical planning. Gender and age impact the healing process and tissue resilience, anatomical factors such as nasal structure and skin thickness dictate the technical nuances of the procedure, and systemic diseases can affect both intraoperative management and postoperative recovery. Consequently, these factors underscore the necessity for individualized patient management, emphasizing a tailored approach to optimize surgical outcomes and address the unique needs and physiological conditions of each patient.

While the study provides valuable insights, the generalizability of findings may be influenced by the heterogeneity of nasal concerns within the cohort. The study primarily focused on immediate postoperative outcomes; a long-term follow-up would offer a comprehensive assessment of the sustained benefits of preoperative nasal CT. Limitations also included the single-center nature of the study. Additionally, the retrospective nature of data analysis was acknowledged as a potential limitation. Future studies with larger, diverse cohorts could further validate the observed advantages.

## Conclusions

In conclusion, the integration of preoperative nasal CT in open structured rhinoplasty represents a transformative approach to surgical planning. The advantages observed in enhanced precision, improved functional outcomes, and individualized aesthetic refinement underscore the potential of preoperative CT as a valuable adjunct in the evolution of rhinoplasty practices. As this study contributes to the growing body of evidence, it encourages the continued exploration and adoption of preoperative nasal CT as an indispensable tool in the pursuit of optimizing precision and patient satisfaction in rhinoplasty.

## Data Availability

Data and material are available upon reasonable request.
